# Injection drug use and HIV/AIDS in China: Review of current situation, prevention and policy implications

**DOI:** 10.1186/1477-7517-3-4

**Published:** 2006-02-01

**Authors:** Han-Zhu Qian, Joseph E Schumacher, Huey T Chen, Yu-Hua Ruan

**Affiliations:** 1 Division of Preventive Medicine, School of Medicine, University of Alabama at Birmingham, Alabama, USA; 2 Department of Health Behavior, School of Public Health, University of Alabama at Birmingham, Alabama, USA; 3 National Center for AIDS/STD Control and Prevention, Chinese Center for Disease Control and Prevention, Beijing, China

## Abstract

Illicit drug abuse and HIV/AIDS have increased rapidly in the past 10 to 20 years in China. This paper reviews drug abuse in China, the HIV/AIDS epidemic and its association with injection drug use (IDU), and Chinese policies on illicit drug abuse and prevention of HIV/AIDS based on published literature and unpublished official data. As a major drug trans-shipment country with source drugs from the "Golden Triangle" and "Gold Crescent" areas in Asia, China has also become an increasingly important drug consuming market. About half of China's 1.14 million documented drug users inject, and many share needles. IDU has contributed to 42% of cumulatively reported HIV/AIDS cases thus far. Drug trafficking is illegal in China and can lead to the death penalty. The public security departments adopt "zero tolerance" approach to drug use, which conflict with harm reduction policies of the public health departments. Past experience in China suggests that cracking down on drug smuggling and prohibiting drug use alone can not prevent or solve all illicit drug related problems in the era of globalization. In recent years, the central government has outlined a series of pragmatic policies to encourage harm reduction programs; meanwhile, some local governments have not fully mobilized to deal with drug abuse and HIV/AIDS problems seriously. Strengthening government leadership at both central and local levels; scaling up methadone substitution and needle exchange programs; making HIV voluntary counseling and testing available and affordable to both urban and rural drug users; and increasing utilization of outreach and nongovernmental organizations are offered as additional strategies to help cope with China's HIV and drug abuse problem.

## Introduction

Illicit drug abuse has become an increasing public health and social concern in the past decades worldwide. Drug abuse causes many problems both to individuals and to societies, including loss of productivity, transmission of infectious diseases, crime, family and social disorder, and excessive health care expenditures [1]. Human immunodeficiency virus (HIV)/AIDS, associated with injection drug use (IDU) and needle sharing to a large extent, has become one of most stunning tragedies in human history. It has caused more than 20 million deaths, and about 40 million people are living with HIV worldwide thus far, with Africa as the most afflicted continent [2]. As the most populous country in the world, China has also observed rapidly increasing drug abuse and HIV/AIDS occurrence in the past 10 to 20 years. China can still shape the course of its epidemic, but it needs to move swiftly and with great resolve [2].

This paper reviews global illicit drug trafficking, drug abuse and its association with HIV/AIDS epidemic in China, and Chinese policies on illicit drug abuse and prevention of HIV/AIDS, and offers additional strategies to governmental crack down on drug smuggling and drug use prohibition to help cope with China's HIV and drug abuse problem. We searched English and Chinese language literature via Medline and the China National Knowledge Infrastructure and reviewed unpublished official data, including national reports on illicit drug control and HIV/AIDS sentinel surveillance data. More than 100 papers and reports were reviewed. Key databases included: (1) Chinese Journal of Epidemiology (*Zhonghua Liu Xing Bing Xue Za Zhi*); (2) Chinese Journal of STD/AIDS Control and Prevention (*Zhongguo Xing Bing Ai Zi Bing Fang Zhi*); and (3) National HIV sentinel surveillance data 1995–2004.

## Illicit drug use in China

Drug abuse in China can be traced to the late Qing Dynasty (1644–1911 A. D.), when British colonists forcefully brought Indian opium into China for exchange of silk, tea, and cash. Opium was then locally planned. By the founding of new China in 1949, more than 20 million Chinese people were opium addicts, representing 5% of the total population [3]. After a short nationwide anti-drug campaign, drug abuse was reported to be eliminated from the mainland in the early 1950s, and for the next three decades China was believed to be a drug-free nation [3].

Illicit drugs reemerged in China in the 1980s as China adopted an open-door policy, and the reemergence was mainly connected with global drug trafficking activities. In Asia, there are two major opiate-producing regions: the "Golden Triangle," comprising three Southeast Asian countries of Myanmar (Burma), Laos and Thailand, and the "Golden Crescent" that includes the three Southwest Asian nations of Afghanistan, Iran and Pakistan [4,5]. The majority of heroin and opium in the current Chinese market is brought from Myanmar into Yunnan Province or from Viet Nam into Guangxi Province, and then it is trans-shipped along inland trafficking routes to Sichuan, Guizhou, Gansu and Xinjiang or to Guangdong, Shanghai and Beijing [6-8]. A small potion of heroin/opium is trafficked into Xinjiang from the "Golden Crescent" [8]. These drugs further penetrate into other provinces. Since the late 1990s, increasing amounts of amphetamine-type-stimulants (ATS) and other chemically related synthetic drugs including amphetamine, methamphetamine and ecstasy have been locally manufactured and consumed in China [4].

The number of drug users documented officially by Chinese public security departments increased from 70,000 in 1990 to 1.14 million by 2004 [9] while the estimated number is 3.5 million [1]. The lifetime prevalence rates of illicit drug use among residents age 15 years or older in high-prevalence Chinese cities increased from 1.1% in 1993 to 1.6% in 1996, and the 1-year prevalence rate increased from 0.9% to 1.2% during this period [10,11].

The main drug of choice in China is heroin. According to a report of the National Narcotic Control Commission (NNCC), 87.6% of drug users abused heroin in 2002 [12]. The abuse of ATS and MDMA (methylenedioxymethamphetamine or ecstasy) has become popular in city night clubs in recent years [1]. In the 1980s, farmers living in rural bordering areas in Yunnan and Guangxi provinces constituted a large fraction of drug users. Since the early 1990s, more and more urban residents use illicit drugs. The majority of drug abusers are young people with a low education level and limited job skills, although a small proportion of urban users regard using drugs as an indicator of "high social class." NNCC data showed that 74% were aged 17–35 years in 2002 [12].

Experimentation (90%), peer pressure (44%), and relaxation (42%) are commonly cited reasons for beginning to use drugs. Initially, drugs were taken primarily through sniffing/snorting (55%) or smoking cigarettes mixed with drugs (38%) [13]. Injection of drugs became increasingly common among drug users, probably as a result of increasing prices of illicit drugs and greater cost effectiveness of injecting to achieve the desired effect. National behavioral surveillance data showed that the median prevalence of IDU among drug users increased from 35% in April 1995 to 49% in April 2004, and median prevalence of needle sharing among IDUs also increased from 26% to 43% during this period [14,15]. "Buying from drug or grocery stores" and "buying from hospitals" were the two most common routes for obtaining injection needles and syringes [16]. Reasons for sharing a needle included: "do not care about anything else when hooked," "thought it had been cleaned up," "difficult to buy or obtain," "only sharing with selected persons," "no money to buy," and "following other drug users" [16]. Besides IDU and needle sharing, other risk behaviors including unprotected commercial sex put drug users and ultimately non drug users at high risk of HIV [17,18]. It is believed that many female drug users sell sex for drugs, and limited published research also provides supporting evidence [18-20]. However, the impact of the interaction between IDU and commercial sex on HIV risk is not clear. Considering the dramatic increase in the commercial sex industry in China, and the potential bridging role of those with dual risk behaviors in transmitting HIV from high risk groups to the general population, research on the interaction of IDU and commercial sex should be given high priority.

## HIV/AIDS epidemic and its association with IDU in China

Since the first AIDS case was detected in 1985 [21], China has cumulatively reported about 100,000 HIV infections by 2004 [22]. Thus far, the HIV/AIDS epidemic is mainly concentrated in specific geographical areas and sub-group populations [23]. IDU has been the largest contributor to the reported epidemic since 1989, when the first HIV outbreak among IDUs was observed in Ruili City of Yunnan Province bordering with Myanmar [24]. All 31 provinces, autonomous regions and municipalities on mainland China reported HIV infections among IDUs by 2002, and 42% of infections in China were estimated to be due to IDU by 2004 [22].

In 1995, only one out of 8 national sentinel sites for IDU detected HIV infections. The detected prevalence was 0.02% [14]. By 1997, 3 out of 22 sites detected positives, and the average prevalence increased to 6.6%. After that, national average HIV prevalence among IDUs varied between 5.4% and 8.2% [15,22]. However, dramatic geographic differences in HIV prevalence have been observed. Yunnan and Xinjiang provinces have the most severe HIV infection rates among IDUs. Other provinces along or close to drug trafficking roads, such as Guangxi, Sichuan, Guizhou, Hunan, and Jiangxi, have moderate epidemics [25]. HIV began to spread rapidly in Yunnan province in the late 1980s and early 1990s [26]. High prevalence among IDUs was first detected in 1998 in Urumqi City (28.8%) and in Yining City (82.2%) of Xinjiang Province and later on in selected sentinel sites for IDU in other provinces [27]. These include Guangxi (>10%, 1998 sentinel data), Jiangxi (14.5%, 2000), Sichuan (16–20%, 2002), Guizhou (17–19%, 2002), and Hunan (15–20%, 2003) [23]. In 2004, 75% of HIV national sentinel sites for IDU detected HIV infection. More than 20% of HIV prevalence was reported in selected sites in Xinjiang, Sichuan, Guizhou, Guangxi and Hunan [22]. Official HIV prevalence rates in other geographic areas remain <10%; these reports may reflect reality or may be due to failure in detection.

At least 7 HIV-1 subtypes (A, B/B', C, D, E, F, and G), and 3 major circulating recombinant forms (CRF01_AE, CRF07_BC, and CRF08_BC) have spread in China [28-31]. However, the most prevalent HIV-1 strains are subtype B' (44%), C (29%, usually CRF08_BC) and CRF01_AE (13%) [30]. Subtype C and B'/B are the main subtypes among IDUs [30,32]. Co-circulating of multiple subtypes of HIV-1 in China implies the possibility of interclade recombination. Characterization of genetic variability of HIV-1 may help track the epidemic, generate subtype-specific immunological reagents, develop vaccines, and choose antiretroviral therapy regimens.

## Chinese policies on illicit drug control and related HIV/AIDS prevention

Drug trafficking and abuse are illegal in China. Offenders may be sentenced to prison if smuggling 10 grams or more of heroin and could receive the death penalty for smuggling more than 50 gram of heroin. In 1990, the "Regulations on Prohibition Against Narcotics" were enacted with three levels of penalty to be applied to drug users. First-time offenders may be fined and/or allowed to go to voluntary detoxification centers, where they receive 10-day methadone treatment managed by the Ministry of Public Health. The cost of treatment is about 2,000 to 5,000 Chinese yuan – a cost considered expensive for many drug users and their families. If drug users who have gone through a voluntary detoxification program are caught again using drugs, they are sent to compulsory rehabilitation centers (CRC) administered by the Ministry of Public Security for 6 to 12 months. Drug users who relapse users after going through CRC are sent to reeducation-through-labor-centers (RELC) administered by the Justice Department for two to three years [33].

In Chinese history, drug abuse and prostitution have been considered "social evils." The Chinese government typically takes "crackdown" measures and tries to eradicate these phenomena. China did achieve a success story in the 1950s. Illicit drug abuse and prostitution were eradicated through national anti-drug and anti-prostitution campaigns [3,34]. However, this success has not been repeated in the past two decades. One possible explanation is that China has expanded its market economy and opened further to the outside world. Under this situation, it seems impossible to completely stem manufacture of highly profitable illicit drugs and import of drugs along China's long porous land borders with drug-producing countries.

Currently, the Chinese government adopts more pragmatic policies and takes measures targeting both the root and surface of the drug abuse problem. The measures targeting the root include continuously cracking down on drug smuggling activities and discouraging new users through anti-drug education campaigns. The Chinese government actively seeks to collaborate with neighboring countries to prevent drug smuggling across borders and to help Myanmar, for example, to reduce opium poppy cultivation by replacing with crop plantation. Chinese mass media have increased anti-drug education to the general population. Anti-drug education has been included in the curricula for primary and secondary school students [9]. The measures targeting the surface include providing drug detoxification and harm reduction services to drug users. China has about 300 voluntary detoxification clinics, 700 CRC, and 200 RELC. Each can accommodate 100 to 3,000 patients [9]. Both western medications and traditional Chinese medicines are used for detoxification, and community therapy has been provided in some heavily affected areas since the late 1990s [9]. The National Working Group for Community-based Methadone Maintenance Therapy was established collectively by the Ministry of Health, the Ministry of Public Security, and the State Food and Drug Administration. Guidelines for methadone maintenance therapy and needle exchange programs were enacted in early 2004. By the end of 2004, there were 34 methadone substitution therapy clinics and 90 clean needle exchange service points across the country [35,36]. The methadone program will be expanded to about 1,000 sites and treat 200,000 drug users over the next 5 years [35].

## Conclusions and recommendations

Whether or not China can shun a generalized epidemic of HIV/AIDS may be largely dependent on how China deals with IDU risk factors and breaks the bridge between IDU and heterosexual transmission. Past experience in China suggests that solely cracking down on drug smuggling and prohibiting drug use can not prevent or solve all illicit drug related problems in the era of globalization. Governmental support, harm reduction programs, voluntary counseling and testing, and utilization of non-governmental organizations are recommended.

### Strengthening government's leadership at both central and local levels

China has a strong central government. Without government support, it would be not imaginable to achieve success in the campaigns against drug use and the spread of HIV/AIDS. Since the late 1990s, the Chinese central government has stepped up HIV/AIDS control efforts, including setting out national policy framework for responding to HIV/AIDS, increasing funding inputs, expanding collaborations with international organizations. The Chinese National Medium-and-Long-Term Strategic Plan for AIDS Prevention and Control (1998–2010) was formulated in 1998 and set one goal that "by 2002, health education on preventing HIV/AIDS and STDs should be carried out at all detoxification centers and re-education centers as well as in 80% of jails..." [37]. The Action Plan (2001–2005) calls for creating "drug-free communities" through drug prohibition education and drug detoxification activities, together with active promotion of healthy life styles and behaviors and harm reduction for drug users.

On the other hand, there is substantial autonomy at provincial level in some areas. Responses to drug use and HIV/AIDS epidemic vary significantly at provincial and lower administrative levels. For example, Yunnan and Guangxi provinces have done far more than other provinces in supporting, implementing, and advocating for harm reduction interventions for IDUs. Some local governments are not fully motivated to confront drug abuse and HIV/AIDS problems. Some government leaders still ignore or even cover up these problems. They are far more interested in economic growth than HIV/AIDS control and wish for their administrative areas to become "economy provinces" or "economy cities" rather than "AIDS provinces" or "AIDS cities," which is believed to be helpful for their career promotion. Advocacy to and support from government leaders at all administrative levels for harm reduction and community-based prevention are needed.

### Scaling up methadone substitution and needle exchange programs

Harm reduction includes many strategies, such as methadone maintenance, needle exchange, dispensing other drugs, and outreach services [38]. Harm reduction has been a controversial issue as compared to abstinence-based philosophies [39,40]. Harm reduction seems to encourage tolerance of social phenomena that are undesirable and hazardous and that may result in social turpitude. Instead, abstinence is considered to be the proper way to address drug problems [39]. In the United States, law and policy restrict the use of federal funds in supporting needle and injection equipment distribution projects. However, many studies refute the concern that access to sterile syringes is an endorsement of IDU and is likely to result in increases in injection and initiation of injection [41,42]. Harm reduction projects in China are in the pilot phases. Some scholars and health officials still have similar concerns as in some Western countries. They believe that needle exchange services may send a wrong signal of encouraging drug abuse to drug users and the public, and they consider methadone substitution unethical because it uses one drug to replace another drug [43]. As drug use and HIV/AIDS spread rapidly across the country, it is urgent to find supporting domestic evidence that harm reduction will reduce HIV transmission by evaluating harm reduction projects and ultimately scaling up harm reduction efforts.

It is easy to access sterile needles and syringes in urban areas of China because they are legally sold and available at pharmacies and medical clinics [16,44]. However, many drug users live in rural areas; in addition, drug users may share needles and syringes because they can not buy them during night time or do not have money to buy them [16]. Methadone is orally administered, and methadone substitution can reduce injection and needle sharing of opiate drug addicts. But methadone maintenance therapy is costly and requires drug users to attend clinics on a regular basis. Therefore, methadone substitution and needle exchange services should be made available and affordable at convenient times and in both urban and rural settings, especially in the communities with heavy drug use.

In addition to cost and availability, other factors might also affect acceptability of methadone maintenance therapy, such as concerns about the safety and efficacy of the therapy [45]. Greater retention in treatment has been found to result in greater decreases in drug use, criminal activity, and unemployment [46]. The length of drug treatment has a positive association with better post-treatment outcome [46]. However, limited experience with methadone maintenance therapy (MMT) in China shows a high rate of dropouts. International studies have shown that motivational enhancement therapy or motivational interviewing enhances treatment initiation, retention and outcomes in MMT program [47,48], and adding behavioral intervention components into MMT programs increases abstinence and reduces HIV risk behaviors [49]. Policy-oriented operational research is needed in China to better understand how to increase the effectiveness of MMT and other harm reduction interventions in the Chinese context.

There are still persistent conflicts in the policy and legal landscape. The central government has given explicit support to harm reduction, for example, as stated in the Medium- and Long-Term Strategic Plan and the Action Plan. Some programs have been implemented successfully [36,50,51]. However, in China, as in many other countries, public health and public security authorities frequently approach drug abuse from different perspectives, leading to conflicting approaches at local levels. The crackdown philosophy and detention of drug users in China reflect inconsistent interpretations of "harm reduction" and present a challenge to public health officials in implementing methadone substitution and needle-exchange programs [52]. Drug users may be reluctant to participate in these programs due to fear of being caught by police officers [50]. It might be impossible to completely solve the dilemma in the near future, but this conflict is expected to gradually reduce for the following reasons. First, Chinese national policies for HIV prevention and control have become much more pragmatic in the past years. MMT and needle exchange programs were almost unimaginable several years ago, but now they are ready to be expanded across the country. We expect that the open policy trend will continue as the Chinese economy is increasingly merged with international markets, and this trend will favor harm reduction programs. Furthermore, China's centralized government may achieve an advantage in promoting public health policies if these policies are believed to be correct. Second, inter-agency coordination on public health crisis has been enhanced at both central and local governmental levels since SARS outbreak in 2003, which reduces potential conflict of public health policies. Public health workers should provide policy advocacy to public security authorities and help them change their traditional norms about illicit drug control and obtain their supports for harm reduction. Third, operational research is needed to provide evidence on the benefits of harm reduction programs and convince policy enforcers and lead to revision of unfavorable policy components.

### HIV voluntary counseling and testing

HIV voluntary counseling and testing (VCT) is often considered the first step for initiating prevention and/or therapy. One of the strategies for addressing the AIDS epidemic is to give people an opportunity to know their HIV status so that they can take precautions to avoid further spread and receive early therapy if they are infected [53,54]. However, even in developed countries, many at-risk people do not take VCT. A national British survey in 2000 showed that only one-third of IDUs had VCT in the past 5 years [55]. About one-fourth of the 0.8 to 0.9 million infected people in the United States remain unaware that they are HIV positive [54]. In China, there is a large discrepancy between reported (about 100,000) and estimated (about 1 million) cumulative HIV/AIDS cases thus far. Many at-risk individuals do not seek out standard HIV counseling and testing services. The stigma associated with drug use and HIV/AIDS and fear of arrest or knowing a positive result can be major barriers to access to VCT. A survey among 840 pregnant women and 780 health professionals in Yunnan Province – an epicenter of the HIV/AIDS epidemic in China – found prevalent negative attitudes toward HIV/AIDS. Twenty-three percent of health professionals and 45% of pregnant women thought HIV was a disease of "low class and illegal" people; 48% of health professionals and 59% of pregnant women thought that HIV positive individuals should not be allowed to get married; and 30% of the health professionals were not willing to treat an HIV-positive individual [56]. Cost of traditional VCT, low awareness of risk factors for HIV infection, distance, and inconvenience in time also may prevent access to VCT. Possible solutions include development of outreach programs to offer anonymous testing and counseling to those at heightened risk of HIV infection and adoption of new technologies such as rapid saliva testing and counseling strategies to improve the outreach and efficacy of programs.

### Outreach and non-governmental organizations

Non-governmental organizations (NGO) can play a critical role in the delivery of HIV prevention services and other assistance to persons living with AIDS. The flexibility of NGOs enables them to respond quickly to fill in gaps in health care and social services. NGOs can do what government agencies cannot do or are not willing to do – for example, reaching out without perceived threat to IDUs and other marginalized sub-groups whose behaviors are often stigmatized and also put them at higher risk of HIV/AIDS. A recent survey of 29 NGOs in Central and Eastern Europe showed that most NGOs targeted injection drug users; provided needle exchange and HIV prevention peer education; and delivered AIDS presentations and distributed educational materials [57]. In Africa, where the main transmission occurs via heterosexual activity, NGOs are most likely to direct their attention to the general public and to youth; they provide peer-education or community outreach [58]. In both Thailand and Brazil, where success has been observed in controlling the HIV/AIDS epidemic or reducing AIDS mortality, NGO are believed to play a key role, but their programs lack rigorous and systemic evaluation [59,60]. NGOs often face several difficulties: lack of financial resources [57,58]; lack of communications with governmental organizations [61]; governmental indifference or opposition; and AIDS-related stigma [57].

China has large number of government organized NGOs (GONGOs), including Family Planning Associations, Women's Federation, Red Cross, Youth League, trade unions, and diverse academic associations. The members in these GONGOs have formal positions in governmental organizations while they volunteer at GONGOs. The "true" NGO that has no relationship to the government is just emerging in China. More and more of existing Chinese GONGOs are getting involved in sexually transmitted disease/AIDS prevention. Since the 1990s, the Chinese government has encouraged them to participate in HIV/AIDS control. These government-sponsored NGOs support HIV/AIDS education and academic publications and participate in AIDS research and education with foreign governmental and non-governmental partners, and they can serve as a powerful aid to the Chinese government to achieve the goal of stopping further spread of drug use and HIV/AIDS epidemic.

In spite of its potential for greatly contributing HIV prevention in China, there is little literature in this area. An exception is a recent study (Chen and Liao, 2005) of a Women Federation's HIV prevention program in south China. The study showed that the Women Federation was able to deliver a culturally oriented, multi-level intervention program targeted at female drug users. The data also indicated that the program was successfully in increasing knowledge about HIV/AIDS, increasing condom use, and decreasing needle and syringe sharing among the female drug users in the project. Studies which systematically evaluate the implementation and effectiveness of NGO based intervention programs are greatly needed in the future.

## List of abbreviations

ATS – Amphetamine-type-stimulants

CRC – compulsory rehabilitation centers

GONGO – government organized non-governmental organization

HIV – human immunodeficiency virus

IDU – injection drug use

MDMA – methylenedioxymethamphetamine

MMT – methadone maintenance therapy

NNCC – National Narcotic Control Commission of China,

NGO – non-governmental organization

RELC – reeducation-through-labor-center

VCT – voluntary counseling and testing

## Competing interests

The author(s) declare that they have no competing interests.

## Authors' contributions

HZQ and JES conceived of the study and wrote the first draft of the manuscript, HZQ, HTC and YHR collected the data, and all authors participated in the data interpretation and manuscript revisions.
